# Supplementary material for: A Likelihood Approach for Real-Time Calibration of Stochastic Compartmental Epidemic Models
Source: PLoS Comput Biol. 2017 Jan 17;13(1):e1005257. doi: 10.1371/journal.pcbi.1005257 (PMC5240920; doi:10.1371/journal.pcbi.1005257)
Supplement: S1 File — (TAR.GZ) [file pcbi.1005257.s014.tar.gz › HSPH_Online-SI-Revision/EnKF07/n100-severe/n100-severe_EnKF_table8.pdf]

1	R0	{1.65278, 9.06793}	{2.13378, 1.88073}	{{0.19862, 0.235621}, {3., 6.70172}}	{83.3948, 79.2596}	inf	1287.	3166.48	{242.507, 4994.3}	168.598	0.384006	newly	875.	3839.5	{532., 7310.}	351.623	0.531196	Reff	0.583613	0.315139	{0.0489959, 0.784868}	64.4191	ar	66342.	165372.	{157267., 181767.}	149.272
2	R0	{1.4321, 3.80644}	{2.21479, 9.97387}	{{0.544503, 1.73899}, {3., 14.0404}}	{94.243, 178.384}	inf	47.	8475.46	{823.174, 12764.4}	17932.9	1.83651	newly	65.	7112.03	{346., 10547.}	10845.2	1.53585	Reff	0.65732	0.57595	{0.0886441, 1.29281}	68.3191	ar	54217.	182011.	{161673., 213072.}	235.709
3	R0	{1.68388, 4.76028}	{2.70248, 8.27896}	{{2.14697, 2.61652}, {3., 12.3848}}	{60.4911, 97.3284}	inf	45.	5061.81	{553.407, 3356.76}	11148.5	1.68112	newly	41.	3937.85	{383., 5902.}	9504.51	1.60866	Reff	0.534935	0.627991	{0.288423, 1.76653}	66.432	ar	68299.	193636.	{174891., 241528.}	183.512
4	R0	{1.66014, 6.95383}	{2.58924, 12.0344}	{{2.06646, 3.49643}, {3., 17.0581}}	{55.9653, 107.509}	inf	535.	6068.92	{1287.95, 14662.1}	1034.38	0.874799	newly	463.	4421.52	{1200., 8423.}	854.972	0.714744	Reff	0.564215	0.657692	{0.179951, 2.06644}	81.3622	ar	66820.	178399.	{156682., 257477.}	166.985
5	R0	{1.69984, 11.2038}	{2.14153, 3.03868}	{{0.388915, 0.637871}, {3., 6.96354}}	{76.5086, 79.8782}	inf	2209.	7834.22	{5297.23, 6166.68}	254.65	0.443206	newly	1193.	12941.7	{3630., 39921.}	984.806	0.874704	Reff	0.580631	0.633606	{0.0645034, 0.980299}	89.216	ar	68462.	176577.	{156411., 222144.}	157.92
6	R0	{1.65876, 6.66217}	{2.73307, 8.69278}	{{2.13892, 0.593212}, {3., 13.5565}}	{64.7656, 88.7366}	inf	667.	7410.42	{1908.36, 4625.46}	1014.83	0.844212	newly	562.	8937.21	{1198., 15867.}	1494.45	0.887633	Reff	0.560711	0.927826	{0.599892, 2.1389}	81.1259	ar	69396.	179999.	{151744., 244501.}	168.235
7	R0	{1.69966, 5.89996}	{2.03008, 11.6862}	{{0.509504, 6.27385}, {3., 14.6646}}	{73.8968, 98.0725}	inf	268.	4890.42	{936.338, 3150.3}	1730.06	1.01052	newly	239.	2592.17	{640., 2318.}	990.473	0.776393	Reff	0.52426	0.380236	{0.0302465, 0.678347}	56.4392	ar	69396.	172022.	{161955., 179693.}	147.885
8	R0	{1.53428, 6.3734}	{1.94348, 10.1642}	{{0.054229, 2.67215}, {3., 19.6251}}	{91.7885, 122.79}	inf	613.	4367.85	{430.561, 6507.8}	621.052	0.65631	newly	560.	3022.2	{502., 9071.}	448.332	0.520463	Reff	0.635852	0.274849	{0.00867735, 0.536104}	57.9213	ar	59855.	168843.	{160722., 180693.}	182.087
9	R0	{1.58939, 3.48608}	{2.08873, 9.78372}	{{1.10058, 3.43819}, {3., 18.894}}	{60.8846, 181.31}	inf	3.	12124.5	{508.809, 28512.1}	404049.	3.23256	newly	4.	7002.55	{518., 14832.}	174964.	2.93461	Reff	0.594845	0.473162	{0.208048, 0.940799}	41.6388	ar	62575.	195022.	{169419., 259194.}	211.66
10	R0	{1.58528, 5.5593}	{2.36923, 4.96339}	{{1.24628, 0.684882}, {3., 15.1117}}	{64.7769, 69.1557}	inf	262.	4778.02	{2827.09, 3471.13}	1726.08	1.09661	newly	240.	7875.8	{1257., 7826.}	3190.4	1.24952	Reff	0.591785	0.580581	{0.0732647, 0.984444}	35.7502	ar	63039.	182944.	{168689., 214075.}	190.208
11	R0	{1.58305, 4.62721}	{1.96911, 12.6498}	{{0.542967, 8.19031}, {3., 14.778}}	{79.4418, 173.379}	inf	119.	3727.69	{1338.79, 2708.99}	3032.52	1.33822	newly	130.	1870.56	{540., 1898.}	1338.89	0.978972	Reff	0.585079	0.40355	{0.0491519, 0.700973}	60.4611	ar	63222.	169568.	{160233., 186860.}	168.211
12	R0	{1.70234, 9.34626}	{2.10048, 2.07617}	{{0.271479, 0.0799137}, {3., 7.05806}}	{78.7578, 77.7861}	inf	1435.	10475.9	{6852.48, 6852.48}	635.08	0.73385	newly	887.	12135.6	{4440., 9140.}	1271.95	0.970486	Reff	0.572735	0.301704	{0.0491907, 0.654553}	63.2306	ar	68213.	171978.	{160104., 189029.}	152.119
13	R0	{1.68556, 9.74954}	{2.14009, 1.16684}	{{0.217635, 0.266663}, {3., 3.68446}}	{80.6494, 88.0319}	inf	1573.	3496.49	{2555.37, 2632.98}	128.068	0.272967	newly	954.	5244.42	{1761., 6265.}	451.608	0.577486	Reff	0.561814	0.31313	{0.0311941, 0.636963}	64.8728	ar	68426.	163940.	{155878., 181474.}	139.587
14	R0	{1.59876, 9.78897}	{1.98569, 2.0263}	{{0.249011, 0.29152}, {3., 7.0104}}	{86.2315, 79.3001}	inf	2224.	4992.11	{3119.08, 3119.08}	133.341	0.249224	newly	1422.	6824.78	{2256., 8081.}	386.376	0.493738	Reff	0.640335	0.35585	{0.039007, 0.701978}	63.5227	ar	63553.	165458.	{156831., 166833.}	160.347
15	R0	{1.43577, 5.12819}	{2.63866, 4.49739}	{{1.99627, 1.61603}, {3., 9.7698}}	{83.78, 66.5642}	inf	365.	4564.31	{1791.34, 9823.72}	1151.62	0.924809	newly	410.	8804.	{933., 28948.}	2049.81	1.01386	Reff	0.682565	0.607107	{0.241474, 1.2452}	51.5378	ar	53259.	173341.	{152959., 231934.}	225.467
16	R0	{1.58866, 8.73191}	{2.31282, 7.61492}	{{1.32081, 2.85888}, {3., 15.9899}}	{59.2465, 64.1355}	inf	1770.	4185.09	{400.089, 4823.53}	167.177	0.340032	newly	1205.	4172.58	{170., 6108.}	284.392	0.541695	Reff	0.609442	0.557142	{0.064883, 0.658092}	31.8372	ar	64349.	176064.	{160749., 211893.}	173.608
17	R0	{1.43012, 3.8163}	{2.08873, 12.5337}	{{0.718511, 5.14914}, {3., 16.239}}	{85.6746, 228.426}	inf	129.	5615.79	{659.134, 12211.6}	4254.99	1.39981	newly	198.	3313.55	{486., 6275.}	1580.08	0.969944	Reff	0.671055	0.532658	{0.0929275, 0.787087}	48.4989	ar	53353.	171084.	{158813., 191779.}	220.664
18	R0	{1.47965, 5.16472}	{2.6441, 12.8409}	{{1.80117, 6.67752}, {3., 17.4623}}	{78.6978, 150.103}	inf	307.	12004.7	{7373.83, 19188.8}	3810.31	1.4588	newly	352.	8281.6	{2548., 13062.}	2252.73	1.14457	Reff	0.658962	0.811628	{0.18098, 2.05116}	72.2635	ar	56086.	194352.	{168446., 266170.}	246.524
19	R0	{1.57191, 5.2314}	{2.47052, 14.6583}	{{1.56851, 9.43046}, {3., 17.9107}}	{57.3051, 180.921}	inf	180.	4854.99	{1668.97, 12244.9}	2597.22	1.30919	newly	174.	2330.11	{776., 5668.}	1242.57	1.00347	Reff	0.584248	0.698038	{0.0335743, 1.56886}	70.5276	ar	63119.	176159.	{153985., 236420.}	179.091
20	R0	{1.54435, 5.32911}	{2.79184, 5.18478}	{{2.37484, 2.96949}, {3., 19.8163}}	{80.7779, 62.9554}	inf	264.	3630.44	{2381.55, 7211.85}	1275.17	1.04786	newly	266.	4282.18	{2257., 5627.}	1509.84	1.06133	Reff	0.594389	0.492073	{0.0843829, 1.38521}	66.1401	ar	61870.	176832.	{163092., 213667.}	185.812
21	R0	{1.57932, 4.90031}	{2.31365, 12.4047}	{{0.546398, 8.32466}, {3., 15.2295}}	{83.0389, 153.142}	inf	136.	3342.7	{1509.99, 2767.04}	2359.43	1.27291	newly	157.	1756.48	{699., 1957.}	1021.76	0.913246	Reff	0.576847	0.403566	{0.0628488, 0.698072}	73.6463	ar	63595.	168762.	{158852., 183359.}	165.369
22	R0	{1.47044, 4.07694}	{2.66808, 6.11405}	{{2.04487, 3.22995}, {3., 8.62756}}	{81.4478, 60.3542}	inf	134.	5796.87	{2758.42, 6681.51}	4226.02	1.59939	newly	178.	7907.43	{2009., 26192.}	4342.38	1.48177	Reff	0.652831	0.651197	{0.179486, 1.7306}	51.2071	ar	55895.	177047.	{157452., 248839.}	216.749
23	R0	{1.64997, 3.34546}	{2.04962, 6.89726}	{{1.20649, 4.81678}, {3., 8.01321}}	{52.6908, 106.168}	inf	4.	4898.38	{107.404, 2299.8}	122359.	2.61883	newly	5.	3527.27	{84., 2533.}	70445.4	2.40893	Reff	0.554456	0.427124	{0.076714, 0.813478}	50.0763	ar	66404.	174665.	{156063., 214405.}	163.034
24	R0	{1.67405, 4.50254}	{2.53071, 6.76878}	{{1.98588, 4.08511}, {3., 10.1993}}	{51.1727, 60.716}	inf	61.	5049.09	{2879.38, 7482.25}	8177.2	1.87301	newly	72.	7135.49	{1584., 23071.}	9810.4	1.78738	Reff	0.536115	0.697193	{0.171437, 1.98586}	70.7447	ar	68017.	182527.	{163784., 251557.}	168.354
25	R0	{1.50663, 3.1286}	{2.62102, 4.64122}	{{2.01071, 3.03075}, {3., 4.42481}}	{73.9657, 51.1005}	inf	23.	2757.12	{1344.03, 1801.35}	11887.5	1.85829	newly	31.	4267.88	{1225., 4726.}	13667.4	1.83652	Reff	0.620536	0.686479	{0.193227, 2.01069}	70.0067	ar	58823.	175513.	{160305., 243671.}	198.374
26	R0	{1.69822, 6.34992}	{2.39715, 7.41841}	{{1.27291, 3.42017}, {3., 11.1499}}	{56.9624, 50.9692}	inf	344.	13185.	{3313.62, 32085.1}	3732.88	1.41861	newly	289.	10058.2	{2222., 28323.}	3380.75	1.39097	Reff	0.518704	0.481619	{0.245483, 0.852392}	42.8327	ar	69816.	190367.	{162402., 261055.}	172.67
27	R0	{1.55869, 4.92988}	{2.58558, 15.183}	{{1.65764, 9.5967}, {3., 17.8663}}	{65.8819, 207.979}	inf	156.	7386.98	{676.172, 5850.13}	4635.24	1.4897	newly	170.	3305.81	{271., 3404.}	1844.59	1.08278	Reff	0.591429	0.617741	{0.083072, 2.02879}	74.8399	ar	62344.	170061.	{151019., 235502.}	172.778
28	R0	{1.46085, 6.06899}	{2.50502, 9.93773}	{{1.39264, 7.25796}, {3., 16.8253}}	{73.72, 63.746}	inf	471.	16213.4	{1524.14, 56992.2}	3344.6	1.18975	newly	430.	10904.8	{986., 38165.}	2438.88	1.01725	Reff	0.667083	0.462096	{0.0714288, 1.28924}	67.846	ar	55484.	191294.	{155435., 271797.}	244.773
29	R0	{1.63017, 6.25164}	{2.62179, 14.0703}	{{1.87873, 9.41428}, {3., 17.4864}}	{60.8293, 125.066}	inf	417.	9302.68	{1628.2, 11466.8}	2130.86	1.24091	newly	364.	4681.38	{670., 5564.}	1186.09	0.990643	Reff	0.557469	0.907168	{0.229287, 1.87932}	85.0733	ar	66231.	191641.	{160050., 244525.}	189.352
30	R0	{1.45173, 5.68165}	{2.28667, 6.08611}	{{1.33949, 0.563089}, {3., 15.2451}}	{64.0673, 80.3486}	inf	490.	4069.3	{1279.27, 3018.92}	731.782	0.679855	newly	473.	3353.93	{746., 5715.}	612.129	0.696511	Reff	0.663223	0.50703	{0.176127, 0.924253}	47.8996	ar	55280.	171808.	{158344., 210733.}	210.797
31	R0	{1.55808, 2.77316}	{2.61006, 6.9637}	{{1.91683, 4.15899}, {3., 10.6816}}	{67.5177, 151.111}	inf	8.	3392.16	{2825.9, 5086.08}	42302.	2.60751	newly	17.	4193.63	{1372., 11247.}	24568.4	2.24271	Reff	0.608757	0.574003	{0.06053, 1.91681}	85.1064	ar	60940.	184939.	{169903., 250371.}	203.478
32	R0	{1.56797, 3.90327}	{2.64656, 6.87135}	{{1.91152, 3.19173}, {3., 8.59893}}	{68.7891, 91.2182}	inf	58.	9290.03	{3659.69, 3659.69}	15917.3	1.88124	newly	67.	6810.34	{2122., 5592.}	10064.7	1.72863	Reff	0.594323	0.441911	{0.0606136, 0.958126}	66.8075	ar	62228.	191844.	{171375., 250664.}	208.293
33	R0	{1.70027, 5.43261}	{2.38554, 5.91397}	{{1.01604, 2.99393}, {3., 6.8923}}	{65.1871, 24.4476}	inf	114.	10381.8	{1400.87, 5247.74}	9006.85	1.65068	newly	106.	8542.68	{1294., 16569.}	7959.13	1.62407	Reff	0.533137	0.443289	{0.138459, 0.638113}	36.7138	ar	69878.	190082.	{169765., 239248.}	176.291
34	R0	{1.61668, 6.86192}	{2.09042, 3.54309}	{{0.24078, 1.94329}, {3., 6.59909}}	{85.3546, 48.366}	inf	584.	8489.75	{5547.09, 7316.42}	1356.81	1.03996	newly	504.	9046.38	{3742., 10954.}	1697.62	1.10141	Reff	0.585885	0.261355	{0.044919, 0.605919}	63.7674	ar	64693.	188236.	{177403., 206124.}	190.968
35	R0	{1.55955, 3.45071}	{2.1603, 6.33592}	{{1.21306, 3.60725}, {3., 8.54498}}	{59.3843, 90.3227}	inf	18.	6962.	{3678.45, 10678.3}	38577.8	2.40533	newly	21.	7489.09	{2221., 23225.}	35562.3	2.30448	Reff	0.596107	0.554462	{0.079346, 1.21329}	47.2317	ar	61783.	184836.	{165497., 230157.}	199.169
36	R0	{1.65068, 4.91307}	{2.22355, 12.11}	{{0.570617, 8.07958}, {3., 14.6272}}	{76.466, 146.485}	inf	177.	2735.61	{862.759, 2820.77}	1445.58	1.05934	newly	195.	1439.42	{361., 2087.}	639.938	0.718335	Reff	0.557286	0.361405	{0.0781704, 0.728773}	55.944	ar	66453.	166809.	{161601., 170451.}	151.018
37	R0	{1.46131, 3.06714}	{2.29257, 6.9011}	{{1.35446, 3.38165}, {3., 8.14347}}	{63.1518, 125.001}	inf	14.	5064.82	{3820.86, 3820.86}	36077.3	2.44344	newly	26.	5271.55	{2245., 5278.}	20175.2	2.0813	Reff	0.636678	0.444999	{0.0863463, 0.620663}	49.2439	ar	56452.	173451.	{157089., 236985.}	207.253
38	R0	{1.71034, 5.2546}	{2.08101, 10.9256}	{{0.445108, 8.12069}, {3., 12.7219}}	{74.8445, 107.924}	inf	137.	5359.82	{1213.5, 8500.92}	3815.31	1.32883	newly	136.	3033.85	{610., 4310.}	2134.21	1.06042	Reff	0.519003	0.292119	{0.0255028, 0.608666}	56.3198	ar	69816.	172633.	{163773., 188576.}	147.268
39	R0	{1.43565, 5.57989}	{2.43787, 9.21811}	{{1.38339, 7.31323}, {3., 17.4054}}	{71.8893, 65.2023}	inf	344.	14541.8	{2452.4, 55151.4}	4127.45	1.32196	newly	352.	9795.69	{1466., 36308.}	2684.43	1.12828	Reff	0.663615	0.502231	{0.180721, 1.12621}	57.2165	ar	54407.	188878.	{160413., 272882.}	247.158
40	R0	{1.40327, 4.00764}	{2.14194, 13.3183}	{{0.676464, 6.82616}, {3., 17.0271}}	{90.7317, 232.324}	inf	152.	6532.66	{350.379, 5063.38}	4204.08	1.44156	newly	210.	3319.2	{197., 4635.}	1492.49	1.04182	Reff	0.680319	0.451985	{0.117672, 0.815835}	46.2498	ar	51976.	167841.	{155947., 170299.}	222.921
41	R0	{1.63612, 4.26741}	{2.29579, 6.63027}	{{1.28208, 3.43422}, {3., 7.82456}}	{58.0466, 65.9128}	inf	56.	3780.59	{3252.63, 3252.63}	6651.05	1.74993	newly	65.	4023.04	{1927., 4307.}	6089.29	1.59577	Reff	0.573476	0.409299	{0.0365976, 0.749752}	56.0099	ar	65029.	172858.	{159078., 226470.}	165.816
42	R0	{1.57426, 6.50271}	{2.6331, 4.45431}	{{2.00838, 1.78901}, {3., 9.66875}}	{67.2597, 59.714}	inf	569.	5624.7	{2037.81, 3863.3}	892.785	0.746173	newly	480.	9039.72	{1129., 39387.}	1787.13	0.910017	Reff	0.602202	0.518538	{0.0917451, 2.00836}	86.723	ar	62522.	181815.	{158275., 245095.}	190.801
43	R0	{1.37818, 5.37636}	{2.33398, 6.40364}	{{1.37376, 0.620509}, {3., 15.9729}}	{69.5213, 87.2364}	inf	520.	8461.16	{3889.96, 26827.4}	1527.15	0.981176	newly	563.	7404.53	{1459., 12789.}	1221.85	0.943559	Reff	0.701742	0.512617	{0.0439006, 1.22913}	47.9628	ar	50098.	179037.	{161891., 223238.}	257.374
44	R0	{1.66606, 4.92641}	{2.69566, 8.11983}	{{1.94296, 3.62987}, {3., 10.4273}}	{61.7987, 82.1925}	inf	140.	5274.09	{701.978, 5699.41}	3670.5	1.42864	newly	138.	6029.65	{550., 6885.}	4272.92	1.32222	Reff	0.551433	0.549779	{0.136208, 0.723007}	45.2661	ar	67066.	180769.	{167350., 184810.}	169.538
45	R0	{1.623, 5.9449}	{2.68944, 9.56542}	{{2.20369, 0.650735}, {3., 13.5318}}	{65.7079, 105.399}	inf	438.	9326.25	{1700.47, 13959.7}	2030.23	1.05851	newly	424.	8600.06	{992., 15267.}	1929.6	1.00098	Reff	0.585189	1.09449	{0.633612, 2.20367}	91.1876	ar	64554.	190351.	{157734., 272586.}	194.872
46	R0	{1.65235, 5.51234}	{2.67665, 13.8285}	{{2.03323, 9.48354}, {3., 18.0138}}	{61.9904, 151.679}	inf	274.	6261.85	{2429.82, 8669.99}	2186.5	1.26824	newly	254.	3117.26	{1050., 5202.}	1128.91	0.965786	Reff	0.558213	0.491647	{0.266608, 0.815809}	36.62	ar	66509.	164446.	{149542., 196761.}	147.254
47	R0	{1.62138, 8.82208}	{2.52683, 3.70255}	{{1.47404, 2.37999}, {3., 4.59258}}	{61.3232, 62.773}	inf	1228.	5317.04	{2062.6, 15334.7}	335.091	0.390992	newly	834.	5507.67	{1983., 16331.}	552.968	0.62024	Reff	0.585561	0.390352	{0.123235, 0.542564}	55.5933	ar	65598.	191110.	{177607., 238864.}	191.335
48	R0	{1.64073, 6.69261}	{2.67951, 9.58585}	{{2.08432, 0.133368}, {3., 14.1549}}	{63.3121, 98.0911}	inf	581.	6424.33	{2443.43, 5896.92}	1008.96	0.903214	newly	486.	8895.	{1288., 17387.}	1733.63	0.918952	Reff	0.561064	0.82911	{0.23683, 2.0843}	86.375	ar	66438.	179086.	{155591., 240202.}	169.554
49	R0	{1.65556, 3.796}	{2.57873, 6.49216}	{{1.99697, 4.07324}, {3., 10.2756}}	{55.7617, 71.0264}	inf	19.	5488.18	{2960.89, 8004.98}	28785.1	2.41209	newly	19.	8204.21	{1699., 22960.}	43080.1	2.42029	Reff	0.553586	0.742865	{0.105064, 1.99695}	86.6247	ar	66255.	189211.	{166046., 262950.}	181.482
50	R0	{1.56828, 3.65713}	{2.31719, 6.71615}	{{1.24901, 3.37979}, {3., 7.95813}}	{63.6234, 87.7404}	inf	15.	4780.21	{3269.32, 3269.32}	31768.	2.36389	newly	19.	5204.2	{1953., 5081.}	27290.5	2.1761	Reff	0.584592	0.435368	{0.0930572, 0.803623}	50.5179	ar	62761.	171640.	{159695., 206492.}	171.482
